# Basal Cell Carcinoma in a Child

**DOI:** 10.1155/2011/752901

**Published:** 2010-09-22

**Authors:** Samet Vasfi Kuvat, Zuhal Gücin, Barış Keklik, Gülzade Özyalvaçlı, Karaca Başaran

**Affiliations:** ^1^Dicle Medical Faculty, Department of Plastic and Reconstructive Surgery, 21280 Diyarbakır, Turkey; ^2^İstanbul Training and Research Hospital, Department of Pathology, 34098 İstanbul, Turkey; ^3^İstanbul Medical Faculty, Department of Plastic and Reconstructive Surgery, 34390 Diyarbakır, Turkey; ^4^Seyitömer Mah. Emrullah Efendi Sok. no. 60/6, Fındıkzade 34098 Fatih, İstanbul, Turkey

## Abstract

Basal cell carcinoma is the most commonly seen nonmelanoma skin cancer which is rarely encountered in the childhood period. An 11-year old child was admitted to our clinic due to an erythematous and a slightly pigmented lesion with a 3 × 4 cm diameter on his posterior scalp. Macroscopically, the lesion was excised with a 10 mm safety margin. Pathologic examination revealed a basal cell carcinoma. No symptoms or signs of a syndrome were observed both in the patient and his family.

## 1. Introduction

Non-melanoma skin cancers appear to be the most often malignant tumors. Basal cell carcinoma (BCC) accounts for 75% of non-melonoma skin cancers and peaks in the seventh decade [1]. Although it is frequent in the elderly, BCC is extremely rare in children under 15 years of age. Basal cell carcinoma seen in the pediatric age group is associated with inherited syndromes like basal cell nevus syndrome, xeroderma pigmentosum, Bazex syndrome, vitiligo, albinism, and congenital lesions like nevus sebaceous. High-dose radiotherapy has been defined as a risk factor as well. Severe or increased sun exposure has been most often implicated in the etiology of nongenetic BCC [2–6]. Ultraviolet rays induce DNA damage leading to overexpression of the oncogens together with the depression of tumor suppressor genes (Sonic Hedgehog and p53) [7]. Other than the ionizing radiation, arsenicals and polyaromatic hydrocarbons play a role in etiology as well [5]. 

## 2. Case

**Figure 1:**
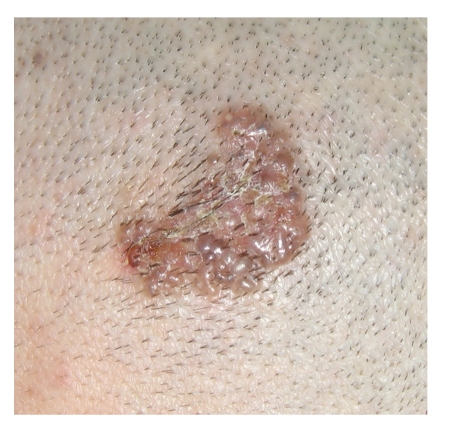
Macroscopic appearance of the lesion on the scalp.

**Figure 2:**
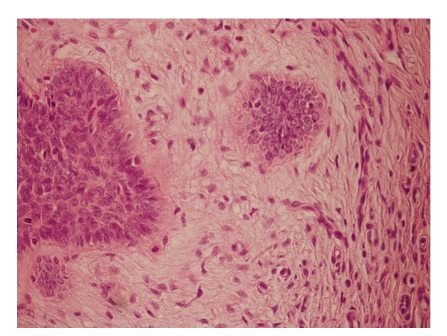
Large magnification showing the intersection area of the normal dermis stroma and the stroma caused by the tumor on the right, and the typical palisadic arrangement of the cells on the peripheral tumor on the left (H and E stain; original magnification: x200).

An 11-year-old boy was admitted to our clinic with the complaint of a lesion of 15-month duration on the hairy scalp. Physical examination revealed an erythematous, slightly pigmented plaque lesion approximately 3 × 4 cm in diameter on the posterior scalp (Figure 1). Excisional biopsy was done with a 10 mm surgical margin. On histopathologic examination, there were typical nodular basal cell carcinoma tumor islands with ulceration localized in the dermis. Asymmetrically growing solid nodules in the dermis with connections to epidermis at some zones were seen. Frequent mitoses were noted as well. Retraction artefact between tumor nodules and the stroma were visualized. Adenoid differentiation and pigmentation were observed, as well (Figure 2). No recurrence was noted during the sixteen-month followup of the patient. No other signs or symptoms of syndromes have been encountered in the patient and his family. Any particular risk factors for BCC development have not been documented. 

## 3. Discussion

In a large study of 36207 children done by De la Luz Orozco-Covarrubias et al. [4], 53 types of skin tumors were demostrated in which 36 were primary and 17 were metastatic. Among these 36 primary tumors, only 5 cases were BCC (13%). The incidence of BCC was only 1.9 in 10,000 patients. In another wide series of 6264 chilhood cancers, 21 primary malignant skin tumors were diagnosed and only 9.5% of them were reported to be BCC [5]. In an article published in 2007, 107 idiopathic BCC cases were reviewed and the head was (90.4%) the most often site. Most common localization was cheek in the head and neck region. The scalp accounted for 14.5% of the cases. In the same study, nodular, adenocystic, follicular nodulocystic, superficial, and typical nonaggresive variants were reported to constitute 80% of all cases. Remaining cases were defined as sclerosing, infiltrating, and basosquamous type of aggressive variants. 18% of recurrence was reported in that study. Any correlation between the histopathologic variant and the prognosis was not mentioned [6]. In the case we presented, localization was the posterior scalp and the histopathologic variant was *nodular*. 

BCC is most commonly seen in patients with light-colored hair and skin. Major risk factors for BCC are increased sun exposure, vitiligo-albinism, immunosuppression (AIDS, drug use due to organ transplantation), and radiation. Genetic factors are important as well [4–6]. Most likely cause in our case was supposed to be sun exposure. Other major risk factors of BCC were not encountered at all.

Gorlin-Goltz syndrome (basal cell nevus syndrome) is an autosomal dominant inherited disorder, which is characterized by multiple basal cell carcinoma, maxillary keratocysts, multiple pits of palms and soles, ectopic calcification of cranial membranes, cysts of jaw, and musculoskeletal malformations [8]. Xeroderma pigmentosum is an autosomal recessive inherited disorder, which is characterized by sun sensitivity, increased freckling, pigmentation, and dryness of sun-exposed skin and increased risk of cutaneous neoplasms (BCC, squamous cell carcinoma, and melanoma) [9]. No family history was observed in our case. In additon, no genodermatosis signs have been seen other than the skin pathologies.

 Nevus sebaceous should be kept in mind for a tumor on scalp localization at infancy, childhood, and puberty periods. Sebaceous glands in nevus sebaceous follow the pattern of normal sebaceous glands during these periods. In pubertal age, nevus sebaceous histologically manifests as large numbers of mature sebaceous glands overlying papillomatous epithelial hyperplasia, small hair structures, and ectopic apocrine glands in dermis. In the presented case, any features of nevus sebaseous or trichoepithelioma could not be seen at all. The lesion was diagnosed as idiopathic form of basal cell carcinoma, both clinically and histopathologically.

 Electrodessication, curettage, topical chemotherapy (5-fluorouracil, methotrexate, bleomycin, and interferon), radiotherapy, radium plate or radon mould might be used to treat lesions. Excision with or without the Mohs micrographic technique is the main treatment of basal cell carcinoma. The Mohs technique is very useful in cosmetically sensitive regions such as the nose, lips, eyelids, and ears. After resection, the defects are generally closed primarily. Nevertheless skin grafts and flaps (range from local flaps to free tissue transfer) may be used to repair for larger defects [10, 11]. 

In view of the information so far known, increase in the childhood BCC incidence in recent years should be ascribed to high-level UV radiation exposure [2]. It is not difficult to estimate that the diseases associated with increased UV radiation exposure will rise in the future. Given that the age of BCC appearance is decreased, this tumor should be considered in the differential diagnosis of childhood skin lesions.
